# Commentary to: Hyperoxemia in postsurgical sepsis/septic shock patients is associated with reduced mortality

**DOI:** 10.1186/s13054-022-03932-2

**Published:** 2022-03-23

**Authors:** Manuel Alberto Guerrero-Gutiérrez, Javier Mancilla-Galindo, Ashuin Kammar-García, Luis Antonio Morgado-Villaseñor, Eder Iván Zamarrón-López, Orlando Rubén Pérez-Nieto

**Affiliations:** 1 Department of Anesthesiology and Bariatric Surgery, Baja Hospital & Medical Center, Tijuana, Mexico; 2 Faculty of Medicine, Universidad Nacional Autónoma de México, Ciudad de México, Mexico; 3 Dirección de Investigación, Instituto Nacional de Geriatría, Ciudad de México, Mexico; 4 Intensive Care Unit, Hospital Christus Muguerza Reynosa, Tamaulipas, Mexico; 5 Intensive Care Unit, Hospital Regional IMSS No. 6, Ciudad Madero, Tamaulipas, Mexico; 6 Intensive Care Unit, Hospital General San Juan del Río, Querétaro, Mexico

There is non-conclusive evidence from systematic reviews of randomized controlled trials highlighting that a perioperative fraction of inspired oxygen (FiO2) ≥ 60% confers a greater mortality risk [1]. In the context of critically ill patients, mostly observational studies have addressed mortality risk in patients managed under hyperoxemia—defined differently as partial arterial pressure of oxygen (PaO_2_) greater than 100 mmHg to 487 mmHg—finding a higher mortality risk in these patients [2]. Potential mechanisms of damage by hyperoxemia have recently been reviewed by Singer et al. [3].

For these reasons, recent findings by Martín-Fernández et al. [4] are noteworthy since they found that patients admitted to the ICU after major surgery who had a PaO_2_ > 100 mmHg for more than 48 h had lower 90-day mortality rates than those with a PaO_2_ ≤ 100 mmHg, being associated with a lower adjusted mortality risk. The famous Sagan standard says that “extraordinary claims require extraordinary evidence.” Unfortunately, we believe that the claims by Martín-Fernández et al. were not supported by extraordinary evidence for several methodological and statistical reasons, most of which have previously been covered by editors of respiratory, sleep, and critical care journals [5].

There were important baseline differences between the hyperoxemia and PaO_2_ ≤ 100 mmHg groups in the study by Martín-Fernández et al. Namely, the PaO_2_ ≤ 100 group had higher APACHE-II (16 vs 14) and SOFA (9 vs 7) scores, chronic respiratory diseases (22.3% vs. 12.5), obesity (18.5% vs 11.1%), respiratory tract infection (32.9% vs. 16.3%), and creatinine levels (1.90 vs 1.51 mg/dl). A strategy to diminish confounding due to these differences could have been to perform a propensity score matching analysis since these baseline differences can have an impact on patient outcomes [6].

One strategy to diminish confounding is multivariable regression adjustment. Unfortunately, the authors only adjusted their models for variables that had a p-value < 0.1 in univariate analyses. This approach is not recommended since it does not adequately control for known confounding variables [5]. Building causal diagrams a priori is a better way of selecting appropriate confounding variables to adjust for. Furthermore, they categorized a discrete quantitative variable (APACHE-II) which, if not categorized, could have resulted in different model results. It is not clear whether the authors verified statistical assumptions before creating their models, which is particularly important since APACHE-II and age were both included for adjustment of the models and are at a high risk of collinearity [7]. Regarding the model election, it is unclear why the authors preferred a logistic regression model over a Cox regression analysis which would have been a more appropriate model to apply since they had time-to-event data.

We were very intrigued since Martín-Fernández et al. reported that patients in both groups had a FiO_2_ of 0.5 with little-to-no variance, without reporting peripheral oxygen saturation (SpO_2_). The authors need to clarify whether all patients at their center are managed with the same FiO_2_ regardless of their SpO_2_. Otherwise, this could suggest that this study was not observational, but instead experimental which would raise important ethical issues. Noteworthy, there is a published thesis associated with the same ethics approval number (PI 20–2070), reporting the same number of patients, but with important differences in selection criteria since there is no mention of patients having met 48 h with the same PaO2 and septic shock with a negative culture is not mentioned as an exclusion criteria [8]. Furthermore, since the statistical methods in both the thesis and the paper were the same, it is not clear why the variables for adjustment in both works were different.

We truly believe that these questions may compromise the findings by Martín-Fernández et al. Thus, we requested the dataset from the authors to perform re-analyses and verify these points. Similar to many data sharing requests, our enquiry was rejected by the authors without clear responses to our worries, reflecting the breach that exists between current data availability statements in medical journals and actual data sharing practices [9].

## Data Availability

Not applicable.
